# Collectanea Medica: Consisting of Anecdotes, Facts, Extracts, Illustrations, &c.

**Published:** 1823-06

**Authors:** 


### COLLECTANEA MEDICA:

CONSISTING OF

### ANECDOTES, FACTS, EXTRACTS, ILLUSTRATIONS, &c.

*Relating to the History or the Art of Medicine, and the Collateral Sciences.*

---

Floriferis, ut apes, in saltibus omnia libant,
Omnia nos, itidem, depascimur aurea dicta.

---

### Art. I.—*Extracts from "Medical Jurisprudence."* By J. A. Paris, M.D. &c. and J. S. M. Fonblanque, Esq.

#### *Of the College of Physicians.*

It does not appear that the professors of physic were in any way classed or incorporated, in England, until the year 1522; although we learn, from the preamble of the charter of Henry the Eighth, as well as from the petition of the 9th of Henry the Fifth, that other countries had long before that period established medical colleges, having considered such a measure not only as necessary for the encouragement of science, but as highly politic for the preservation of the public health.

England, although destined to take the lead in research and discovery at a later period, was in the sixteenth century far behind her continental neighbours in the field of science. And, with respect to the study and practice of physic, it seems probable that, until after the foundation of the College of Physicians, it had not even assumed the character and dignity of a regular profession; for we find that the very few learned men in that branch, which the annals of the period can furnish, had acquired their knowledge in the foreign universities.

Until the auspicious period of the Reformation, various circumstances contributed to retard the progress of medical science; the first and most considerable of which may be traced to the many monastic establishments with which the country was infested. The monks are known to have practised physic very extensively; and, when the superstitious character of these ages is considered, we shall not feel surprised at the vulgar, and perhaps not the lower order alone, having preferred, to every other medical assistance, the aid of those who arrogated to themselves the immediate assistance of heaven in the preparation and administration of their medicines.

The alchemists were another, and very numerous class, to whom we may justly refer the temporary degradation of the science of medicine. Like their lineal descendants, the empirics of modern times, their attention was directed to the discovery of an universal specific, which should be equally applicable to every disease; and, as presumption is ever proportionate to incapacity, we need not be surprised that they should have been eagerly followed by the ignorant of their day, as their successors are by the vulgar of our own. Under such circumstances there could have been but little encouragement to men of real learning; and, as we find by the recital of the Act of 5 Hen. VIII. c. 6, that there were but twelve regular surgeons practising in all London, we may safely conclude that the number of legitimate physicians must have been proportionally smaller. The universities of Oxford and Cambridge had probably, from the time of their foundation, conferred degrees in medicine, but these do not appear to have carried with them any general privilege or authority: their rights, indeed, were reserved by the concluding section of the 3d Henry VIII. c. 11; but in what those rights consisted has not been judicially determined, even though the litigation to which the Act and the subsequent charter of the College gave rise would naturally have produced some decision on this point, had the extent of those ancient rights ever been legally defined. We shall not consume any farther time upon this question; for, although it might be a subject of some antiquarian curiosity, it would furnish but little matter of professional interest or practical utility.

In the present age, the universities of Oxford and Cambridge are firmly united by a communion of sentiment and interest to the College of Physicians, and physicians are rarely admitted as fellows of this learned body, unless they have previously graduated in one of the English universities, or at Trinity College, Dublin; but, even in this latter case, it is required that the candidate for admission should have been previously incorporated either into the university of Cambridge or Oxford. That a distinction founded on such a basis should have excited an angry and jealous feeling in the excluded party, is not extraordinary; and the authors of the present work hope that they shall stand excused for offering a few remarks upon a subject which they consider vitally interwoven with the best interests of the profession. The arguments which have been so repeatedly urged against the justice, as well as policy, of the bye-law, which thus excludes all but the graduates of an English university from the honours of the fellowship, may be easily refuted; and its salutary tendency, in relation to the interests of the public, as well as to the dignity of the profession, very satisfactorily demonstrated. For the complete knowledge of medicine, as a science, all the collateral lights of natural philosophy and erudition are required; while, for its successful practice as an art, the physician should possess those high qualifications of mind, and have received that moral cultivation which a mere technical education can never bestow. We are willing to admit that "the curative art cannot be learnt on the sequestered banks of the Cam or the Isis, as well as amid the distress and sickness of a great city;" but we assert, with equal confidence, that the liberal pursuits and wholesome discipline of an English university can best prepare the mind for the full and extensive benefits which the pupil is afterwards to derive from his professional studies in the metropolis; and, if it be essential to encourage a liberal education amongst those who are destined to move in the higher walks of physic, we would ask whether any plan could be devised more likely to ensure our object, than that fair and honourable reward which is held out by this unjustly reviled bye-law of the College of Physicians.

It has been urged, that the education of a physician is thus rendered materially and unnecessarily expensive; and that the delay of twelve years, which are required for the full completion of the highest medical degree, proves another great and vexatious hardship. To all this we reply, that we should politically resist any measure that had the least tendency to divest medical education of its pecuniary sacrifices, and to open the temple to a crowd of needy and half-educated adventurers. Tissot seems to have entertained the same sentiment; and he observes that, for these reasons, no person ought to be allowed to study physic in his native city. The operation of this bye-law will therefore furnish the surest guarantee of professional respectability, and the College of Physicians will continue to enrol names distinguished for science and erudition,—men who will cast a lustre on the profession over which they preside. Let, then, the practitioner in medicine beware how he attempts to depreciate the dignity and importance of this ancient institution, or to deny the rights and privileges to which the corporate body is legally and morally entitled; for to the College of Physicians, as it regards the whole profession of physic, we may address the same emphatic words that Cicero applied to Torquatus with reference to the state, "Tibi, nullum periculum esse perspicio, quod quidem sejunctum sit ab omnium interitu."

Nor is the College singular or invidious, as may at first sight appear, in adopting this rule: by far the greater number, if not all, of the bishops require a similar qualification for the Church; and the Inns of Court, though they do not exclude others, grant some indulgence to members of the university on entering their respective societies, and remit two years of the usual term of probation to those who have taken the degree of Master of Arts or Bachelor of Laws previously to their call to the Bar.

The College of Physicians in London owes its foundation to Dr. Thomas Linacre, of All Soul's, Oxford, one of the physicians to King Henry the VIIIth, a man of profound learning, and most devotedly attached to his profession. Having studied at Rome, Bologna, and Florence, (then under the government of Lorenzo de Medici, by whom he was encouraged,) he naturally imbibed an admiration of the medical schools with which Italy then abounded; and appears to have distinguished himself so much, both by his general learning and particular science, that he was called to court as physician to the king, and entrusted by Henry the VIIth both with the health and education of his son Prince Arthur.

The following may now be considered as the legal classes of physicians:—1st. The actual members of the College of Physicians, divided into their several denominations of President, Elects, and Fellows.

2d. Those who, being graduates of the universities of Oxford and Cambridge, are licensed to practise by the college in London and within seven miles during their respective periods of probation, previous to becoming Fellows: these are candidates who, being doctors of physic, have undergone their examination for the fellowship, and at the end of one year are capable of becoming members or fellows of the college; and inceptor candidates, who, being bachelors of physic, aspire to the fellowship.

3d. The medical graduates of our two universities.

4th. The Licentiates who are admitted by the college to practise in London and within seven miles; and the extra Licentiates, who are admitted to practise in the country, but not within the privileged district of the college.

### *Of the College of Surgeons.*

The present College of Surgeons owes its existence to the Act of the 18th Geo. II. c. 15, by which the surgeons of London are separated from the barbers, with whom they had been made one company and body corporate, by the 32d Hen. VIII. c. 40, previous to which period (A. D. 1540,) the surgeons had no incorporation. They had, indeed, petitioned for and obtained an Act of Parliament under the name of the Wardens and Fellowship of the craft and mystery of Surgeons enfranchised in London, stating their number not to exceed twelve persons, to which number the relief from "quests and other things" granted by the Act is limited; but it is evident, by the preamble to the 32d Hen. VIII., that they, though called a company, "be not incorporate, nor have any manner of corporation," previous to that period. The examination of surgeons, as that of physicians also, had been confided to the bishops; nor does it appear that the subsequent Act of Henry remedied this defect. By the 18th Geo. II., however, they have been made a separate and distinct body corporate and commonalty, under the name of Masters, Governors, and Commonalty of the art and science of Surgeons of London, by which name they may sue and be sued.* All liberties, privileges, franchises, powers, and authorities, which they might have enjoyed under the united company and their Act of Parliament, or under the letters patent of Charles the Ist, or the royal grants, charters, and patents, therein mentioned and referred to, so far as they relate to the science of surgery, are confirmed to them. Now the charter of Charles the Ist, as recited in the preamble of this Act, grants that "no person or persons whatsoever, whether a freeman of the said society or a foreigner, or a native of England, or an alien, should use or exercise the said art or science of surgery within the said cities of London and Westminster, or either of them, or within the distance of seven miles of the said city of London, for his or their private lucre or profit, (except such physicians as are therein mentioned,) unless the said person or persons were first tried and examined in the presence of two or more of the masters or governors of the mystery and commonalty aforesaid for the time being, by four or more of the said examiners so to be elected and constituted as aforesaid, and by the public letters testimonial of the same masters or governors, under their common seal approved of, and admitted to exercise the said art or science of surgery, according to the laws and statutes of the kingdom of England, under the penalty in the said letters patent mentioned." The same charter provides, "That no one, whether a freeman of the mystery or commonalty aforesaid or a foreigner, whether a native of

---

* These names have recently been changed to President, Vice-Presidents, &c.—Editors.

England or an alien, exercising the art of surgery within the cities of London and Westminster, or the suburbs or liberties thereof, or within seven miles of the said city of London, shall go out from the port of London, or send out any apprentice, servant, or other person whomsoever, from the said port, to execute or undertake the place or office of a surgeon for any ship, whether in the service of the crown or of any merchant or others, unless they and their medicines, instruments, and chests respectively, were first examined, inspected, and allowed, by two such masters or governors of the mystery and commonalty aforesaid for the time being, as were skilled, knowing, and professors in the same art of surgery, under the penalty therein mentioned. And the Act, following the same principle, enacts, "That from and after the said 1st day of July, 1745, the examiners of the Company of Surgeons established by this Act shall, and they are hereby required, from time to time, upon request to them made, to examine every person who shall be a candidate to be appointed to serve as a surgeon, or a surgeon's mate, of any regiment, troop, company, hospital, or garrison of soldiers, in the service of his Majesty, his heirs or successors, in like manner as they do, or shall, examine any surgeon or surgeons to be appointed to serve on-board any ship or vessel in the service of his Majesty, his heirs or successors."

By section 3, the College is empowered to hold courts and assemblies, and to make bye-laws, ordinances, rules, and constitutions, for the government of the corporation; and those of the united company concerning surgery are declared to be in force till repealed. By section 11, it is provided that nothing contained in the Act shall abridge or infringe any of the privileges, authorities, &c. of the College of Physicians.

These are the Acts of Parliament which at present regulate the profession of a surgeon: it is evident that they are imperfect, as they do not give any power to restrain and punish ignorant pretenders, who, without the slightest qualification, assume this dangerous and difficult branch of practice, and most especially in the country. We are aware that any attempt of the medical corporations to obtain an increase of their power would create much outcry among those who are interested in the perpetuation of existing abuses: but we will hope that the public safety will be preferred to the private views of empirics; and that a due system of examination, license, and restriction of surgical practise throughout England, will shortly receive the sanction of the legislature.

*Of the Society of Apothecaries.*

In 1606, the apothecaries and grocers were united in one company by charter of the 4th of James the First; but this union did not long continue, for in 1615, by charter of the 13th of the same king, the companies were again separated. This was done upon the representation of some of the apothecaries, backed by the approval of Doctors Mayerne and Atkins, then the king's physicians, by whose interest and solicitation this new charter appears to have been obtained.

This charter was lately confirmed (except as therein altered) by Act of Parliament, 55 Geo. III. c. 194. By this statute, the company's former power of search for unwholesome medicines is repealed; and, in lieu thereof, the master, wardens, and society of apothecaries, or any of the assistants, or any other person or persons properly qualified to be by the master and wardens assigned, not being fewer in number than two, shall, as often as to the said master and wardens may seem expedient, enter, in the day-time, any shop of any person using the art and mystery of an apothecary in any part of England or Wales, and search if the medicines, simple or compound, wares, drugs, or any thing whatsoever therein contained, and belonging to the art or mystery of apothecaries, be wholesome, meet, and fit for the cure, health, and ease of his Majesty's subjects; and all and every such medicines, wares, drugs, and all other things belonging to the aforesaid art, which they shall find false, unlawful, deceitful, stale, unwholesome, corrupt, pernicious, or hurtful, shall and may burn, or otherwise destroy, reporting the name of the offender to the master, warden, and assistants, who may impose and levy on such person the following fines: for the first offence £5.; for the second offence £10.; and for the third and every other offence the sum of £20. No person to be nominated to search drugs, or chosen to the court of examiners, within the city of London or thirty miles of the same, unless he be a member of the Society of Apothecaries, of not less than ten years' standing; nor in any other part of England and Wales, or to be one of the five apothecaries hereinafter mentioned, except he shall have been an apothecary in actual practice for not less than ten years previously to his being so nominated or appointed. "And whereas it is the duty of every person using or exercising the art and mystery of an apothecary, to prepare with exactness and to dispense such medicines as may be directed for the sick by any physician, lawfully licensed to practise physic by the president and commonalty of the faculty of physic in London, or by either of the two universities of Oxford or Cambridge: therefore," if any person using the mystery of an apothecary shall refuse to compound or administer, or deliberately or negligently, falsely or unfaithfully mix, compound, or administer, any medicines ordered by any lawful physician, by any prescription signed with his initials, such person, on complaint made within twenty-one days by such physician, and upon conviction of such offence before any of his Majesty's justices of the peace, unless such offender can show some satisfactory reason, excuse, or justification in his behalf, forfeit for the first offence £5., for the second offence £10.; and for the third offence he shall forfeit his certificate, and be rendered incapable in future of using the art and mystery of an apothecary, and shall be deemed incapable of receiving any fresh certificate until he shall faithfully promise and undertake, and give good and sufficient security, that he will not in future be guilty of the like offence. The master and wardens may from time to time appoint deputies to act for them. The master, wardens, and society of apothecaries, are appointed to carry this Act into execution throughout England and Wales; but no act of the master, wardens, &c. shall be valid, (except the search of drugs, &c. as before mentioned, the acts of the court of examiners, and of the five apothecaries hereinafter mentioned,) unless the same be done at some meeting to be holden in the hall of the society. The powers granted to the master, wardens, and society, to be exercised by the master, wardens, and assistants for the time being, or the major part of them present: the number present at such assemblies not to be less than thirteen, of which the master must be one. Twelve persons properly qualified shall be chosen and appointed by the master, wardens, and assistants, (who may also remove or displace them from time to time, as they may deem advisable;) and such twelve persons, or any seven of them, shall be and be called the Court of Examiners of the Society of Apothecaries, and shall have full power to examine all apothecaries and assistants to apothecaries throughout England and Wales, and to grant or refuse certificates: this court is to meet once a-week at the Hall, a chairman to be appointed, who, in case of equality (his own vote included), shall have a casting vote. The master, wardens, or court of assistants, are to administer a prescribed oath of office to the examiners. The examiners remain in office one year, (except in cases of removal,) and may be re-appointed: in case of death, the successor remains in office only to such time as his predecessor would have done. After the 5th of August, 1815, it shall not be lawful for any person (except persons already in practice,) to practise as an apothecary in any part of England or Wales, unless he shall have been examined by the said court of examiners, and have received a certificate of his being duly qualified to practise as such: no person to be admitted to examination until he shall have attained the full age of twenty-one years; nor unless he shall have served an apprenticeship of not less than five years to an apothecary, and shall produce testimonials, to the satisfaction of the court of examiners, of a sufficient medical education, and of a good moral conduct. Persons intending to qualify are to give notice to the clerk. It shall not be lawful for any person (except persons then acting as assistants, and except persons who have actually served an apprenticeship of five years to an apothecary,) to act as assistant to any apothecary in compounding or dispensing medicines, without undergoing an examination by the court of examiners, or by five apothecaries hereinafter mentioned, and obtaining a certificate of his qualifications.

### *Of Actions by Medical Practitioners.*

A physician cannot maintain an action for his fees, for they are honorary, and not demandable of right: "and it is much more for the credit and rank of that body (the physicians), and perhaps for their benefit also, that they should be so considered; and I much doubt, says Lord Kenyon, whether they themselves would not altogether disclaim such a right, as would place them upon a less respectable footing in society than that which they at present hold." *Chorley* against *Bolcot*, 4 T. R. 317. It was contended in this case, that there was no authority in the books for placing physicians and barristers' fees on the same footing; the regulation with regard to barristers being founded on grounds of public policy, as appears by a passage in Tacitus, to which Mr. Justice Blackstone refers; in which passage it is taken for granted that Medici were entitled to a remuneration, because their situation was dissimilar to that of advocates.

But, though a physician cannot recover his fees by process of law, yet *pro concilio impenso et impendendo* is a good and valuable consideration for an annuity; (9 Co. Rep. 50; 7 Co. Rep. 10, 28.) And this was formerly a very frequent mode of remuneration for professional services both in law and physic, though at the present day it does not frequently occur.

If a bond, bill, or note were given for medical attendance, the consideration would be good, though the original fees could not have been recovered. A distinction might, we think, be drawn between the fees of a physician and his travelling-expenses, which are frequently considerable: but the case of Chorley and Bolcot, before cited, is against it.

If a medical practitioner passes himself off as a physician, (by no means an unfrequent practice in distant parts of the country,) although he has no diploma, and no right to assume that character, he cannot maintain an action for his fees. *Lipscombe* v. *Holmes,* 2 Camp. 441. Though as a surgeon he might have recovered compensation: and, even if he were no regular surgeon, the doctrine in *Gremaire* v. *Le Clerc Bois Valor,* (2 Camp. 144,) would entitle him to recover in an action of assumpsit. But query the authority of this case.

If there be any promise, a physician may receive on a *quantum meruit*; *Shepherd* v. *Edwards*; Hill, 11. Jac. 2. Croke, 370. In this case the plaintiff declared that he, being a professor of physic and surgery, had cured the defendant of a fistula; and he had judgment. All physicians may practise surgery, (32 Hen. VIII.) though surgeons may not encroach in physic: therefore query whether in this case the plaintiff did not sue as a surgeon; and the disease was one which in this day would clearly be classed as a surgical case. It was not so, however, in *Dale* against *Copping,* (Bulst. part 1, p. 39,) when the promise of an infant to pay a certain sum to the defendant for curing him of the falling sickness was held binding; "for that this shall be taken as a contract, and that to be for a thing in the nature of necessity to be done for him, and the same as necessary as if it had been a promise by him made for his meat, drink, or apparel; and in all such cases his promise is good, and shall bind him."

### *Of Actions against Medical Practitioners.*

If a physician, surgeon, apothecary, or other medical practitioner, undertakes the cure of any wound or disease, and by neglect or ignorance the party is not cured, or suffers materially in his health, such medical attendant is liable to damages in an action of trespass on the case: but the person must be a common surgeon, or one who makes public profession of such business, as surgeon, apothecary, &c. for otherwise it was the plaintiff's own folly to trust to an unskilful person, unless such person expressly undertook the cure, and then the action may be maintained against him also. See *Bull*, N. P. p. 73; 2 *Esp.* N. P. p. 601.

" And it seems that any deviation from the established mode of practice shall be deemed sufficient to charge the surgeon, &c. in case of any injury arising to the patient." See *Slater* v. *Baker and Stapleton*, 2 Wils. 359; which was a special action on the case against a surgeon and an apothecary, for unskilfully disuniting the callous of the plaintiff's leg after it was set, which it appears was done for the purpose of trying a new instrument. The plaintiff recovered £500. against the defendants jointly, and the chief justice said he was well satisfied with the verdict. On a motion for a new trial, the judgment was affirmed by the whole court.

In *Seare* against *Prentice*, 8 East's R. 348, it was determined that this action lies against a surgeon for gross ignorance and want of skill in his profession, as well as for negligence and carelessness, to the detriment of a patient; though, if the evidence be of negligence only, which was properly left to the jury, and negatived by them, the court will not grant a new trial, because the jury were directed that want of skill alone would not sustain the action.

In the case of *Neale* v. *Pettigrew*, a surgeon was held responsible in damages for the negligence and unskilfulness of his apprentice or servant.*

Though the cited cases are surgical, there is no doubt that similar actions would be maintainable against physicians or other medical practitioners; but, as internal injuries are less demonstrable than external, there might be some difficulty in obtaining the necessary evidence.†

---

* This case is recent, but we believe not reported. The plaintiff was a respectable artisan, and had been employed as engineer and brass-founder in a large manufactory in the city, and by his industry was enabled to earn about four guineas per week: the plaintiff's right arm was dislocated by a fall from a gig. Mr. Pettigrew, the defendant, was sent for; but, being unable to attend from illness, his assistant undertook the case, but conducted it so unskilfully that the plaintiff lost the use of his arm.—Damages, £800.

† Most of the references are omitted in these extracts.

[To be continued.]

